# Two years study of prevalence and antibiotic resistance pattern of Gram-negative bacteria isolated from surgical site infections in the North of Iran

**DOI:** 10.1186/s13104-020-05223-x

**Published:** 2020-08-14

**Authors:** Hossein Hemmati, Meysam Hasannejad-Bibalan, Sara Khoshdoz, Parisa Khoshdoz, Tofigh Yaghubi Kalurazi, Hadi Sedigh Ebrahim-Saraie, Soheila Nalban

**Affiliations:** 1 Razi Clinical Research Development Unit, Razi Hospital, Guilan University of Medical Sciences, Rasht, Iran; 2 Department of Microbiology, School of Medicine, Guilan University of Medical Sciences, Rasht, Iran

**Keywords:** Surgical site infection, Gram-negative bacteria, Antibiotic resistance, MDR

## Abstract

**Objective:**

The present study aimed to investigate the frequency and antibiotic susceptibility pattern of Gram-negative bacteria (GNB) isolated from surgical site infections (SSIs) in the North of Iran.

**Results:**

This cross-sectional study conducted over a two-year period during 2018–2020 on all cases of SSIs who had a positive culture for a GNB. Standard microbiological tests were followed for the bacterial isolation and identification. Antimicrobial susceptibility profiles were determined using disk diffusion method. During the study period, a total of 78 nonduplicated GNB isolated from SSIs. The most prevalent surgical procedures were fracture fixation (37.2%), and tissue debridement (23.1%). *Klebsiella* isolates showed the highest isolation rate (29.5%) followed by *Enterobacter* (28.2%), and *Acinetobacter* (16.7%). Antibiotic susceptibility results showed that *Acinetobacter* isolates were almost resistant to all of the tested antibiotics, except gentamicin, co-trimoxazole, and meropenem. *Enterobacteriaceae* isolates showed the lowest resistance against amikacin, co-trimoxazole, and imipenem. Overall, 49 (62.8%) of isolates were multiple drug-resistant (MDR). In summary, a remarkable rate of MDR isolates which showed an increasing trend during recent years is a serious alarm for the management of SSIs caused by GNB. Moreover, the results of regional assessments, provide good epidemiological background for comparing our situation with other regions.

## Introduction

Surgical site infections (SSIs) are a devastating complication of hospitalization and one of the global health problems [1]. These infections may involve only the skin and subcutaneous tissues or deep infections involving organs or body spaces [2]. SSIs remain a major cause of morbidity and mortality, impose a high cost on the healthcare system. SSI is the leading health-care-associated infection in the developing countries which also has a markedly higher incidence compared to developed countries [3, 4]. Approximately, the SSI incidence is 5.6 per 100 surgical procedures in developing countries, while in the USA and different European countries the incidence was 2.6 and 2.9 per 100 surgical procedures, respectively [5–7].

The majority of SSIs caused by opportunistic pathogens that originate from the patient’s endogenous microflora. Staphylococci, enterococci, *Pseudomonas aeruginosa*, *Acinetobacter baumannii*, *Enterobacteriaceae*, and *Candida albicans* are the most frequently isolated pathogens in SSIs [5]. However, the epidemiological studies indicate the heterogeneous nature of these infections and the incidence varies widely between procedures, wound classes, and regions [8].

Recently, the emergence of multiple drug-resistant (MDR) strains of hospital pathogens become a global challenge for clinicians [9, 10]. Consequently, the increase of hospital stays, readmissions, additional use of antimicrobials, and treatment failure was expected for patients infected by MDR strains [11]. The rapid rise of MDR Gram-negative bacteria (GNB) is of particular concern since higher mortality risk of nosocomial infections caused by MDR vs. non-MDR GNBs was noted [12, 13].

Given pathogen-specific differences and the many variables associated with adverse outcomes of SSIs, surveillance data about trends in etiology and antimicrobial resistance is a rational way to overcome the risk of drug resistance and treatment failure. Therefore, the present study aimed to determine the prevalence and antibiotic resistance pattern of GNB isolated from SSIs in North Iran. This information can help clinicians to choose effective empirical therapies and provide good epidemiological profiles to compare our situation with others.

## Main text

### Methods

#### Study design and subjects

This retrospective cross-sectional study was conducted on patients with SSIs for two years from March 2018 to March 2020 in a central medical and educational hospital in north Iran. According to Centers for Disease Control and Prevention (CDC) recommendation, SSI was defined as infection occurs within 30 days after the operative procedure and involves only skin and subcutaneous tissue of the incision and organisms isolated from an aseptically obtained culture of fluid or tissue from the superficial incision [14]. During the study, demographic and clinical information of patients who had an SSI caused by GNB were included. The included clinical information consists of surgical procedures, underlying diseases, hospitalization time, hospitalized ward and outcome of patients’. This study was approved by the Ethics Committee of the Guilan University of Medical Sciences and was in accordance with the declaration of Helsinki. However, the committee waived the need for informed consent, because we only used medical records. Also, all personal details of patients were kept strictly confidential.

#### Microbiological procedures

Using aseptic conditions samples either swabs or aspiration were obtained from patients and immediately transferred to the microbiology laboratory. Standard microbiological methods were used for the isolation and identification of the GNB. Briefly, wound samples inoculated to blood, and MacConkey agars and incubated aerobically at 37 °C for 24–48 h. Then, all GNB were identified based on routine microbiological procedures including reaction in Triple Sugar Iron agar, Simmons' citrate agar, Christensen’s urea agar, Indole test, Methyl red and Voges-Proskauer tests [15]. Antimicrobial susceptibility testing against locally available antibiotics was carried out by the disk diffusion method on Mueller–Hinton agar (Merck, Germany) according to the recommendations of the Clinical and Laboratory Standards Institute (CLSI) [16]. The selection of antimicrobial disks (MAST, UK) and interpretation of results for each pathogen was based on CLSI recommendation. *Escherichia coli* ATCC 25922 and *Pseudomonas aeruginosa* ATCC 27853 strains were used for quality control purposes. MDR defined as non-susceptible to ≥ 1 agent in ≥ 3 antimicrobial categories [17].

### Statically analysis

The analysis was performed by using SPSS™ software, version 21.0 (IBM Corp., USA). The results are presented as descriptive statistics in terms of relative frequency. Values were expressed as the mean ± standard deviation (continuous variables) or percentages of the group (categorical variables). Chi-square or Fisher’s exact tests were used to determine the significance of differences. P-value less than 0.05 was considered as a cut off point for statistically significant.

### Results

During the study period, a total of 3781 surgical producers were performed in studied hospital, of which 78 (2.1%) nonduplicated GNB isolated from SSIs. Out of the 78 culture-positive cases, 24 (30.8%) belonged to females and 54 (69.2%) to male cases. The mean age of the patients was 49.0 ± 19.6 (Mean ± SD) years with an age range from 10 to 85 years. The majority of patients were hospitalized in the orthopedic ward (37.2%) followed by neurosurgery intensive care unit (ICU) (26.9%), and general ICU (11.5%). The most prevalent surgical procedures were fracture fixation (37.2%), and tissue debridement (23.1%). The full clinical characteristics of the studied subjects presented in Table 1. Diabetes and hypertension were the most common underlying diseases among studied patients (Additional file 1). Among the 78 cases, the outcome of 7.7% of patients was death, while 92.3% of patients were survived. The overall hospitalization time and hospital stay after an SSI among the studied patients were 16.6 ± 14.6 days, and 13.8 ± 12.2 days, respectively.

**Table 1:** Clinical characteristics of studied cases

Variable	Outcome	Frequency	Percent
Gender	Male	54	69.2
	Female	24	30.8
Ward	Orthopaedic	29	37.2
	Neurosurgery ICU	21	26.9
	General ICU	9	11.5
	General surgery	7	9.0
	NICU	6	7.7
	Neurology unit	3	3.8
	PICU	3	3.8
Surgical procedure	Fracture fixation	29	37.2
	Tissue debridement	18	23.1
	Craniotomy	10	12.8
	Amputation	7	9.0
	Laceration repair	4	5.1
	Laminectomy	3	3.8
	Cranioectomy	2	2.6
	Abdominoplasty	1	1.3
	Cystostomy	1	1.3
	Cranioplasty	1	1.3
	Gastric perforation	1	1.3
	Lumbosacral surgery	1	1.3
Age (year)	Mean ± SD	49.0 ± 19.6	
	Range	10–85	
Overall hospital stay (day)	Mean ± SD	16.6 ± 14.6	
Hospital stay after SSI (day)	Mean ± SD	13.8 ± 12.2	

*ICU* intensive care unit, *NICU* Neonatal intensive care unit, *PICU* pediatric intensive care unit, *SSI* surgical site infection

Regard to bacterial etiology of SSIs, *Klebsiella* isolates showed the highest isolation rate (29.5%) followed by *Enterobacter* (28.2%), and *Acinetobacter* (16.7%) (Table 2). Antibiotic susceptibility results showed that *Acinetobacter* isolates were almost resistant to all of the tested antibiotics, except gentamicin, co-trimoxazole, and meropenem. *Enterobacteriaceae* isolates as the predominant etiology of SSIs showed the lowest resistance against amikacin, co-trimoxazole, and imipenem. Table 3 shows the resistance patterns of isolated pathogens. In overall, 49 (62.8%) of isolates were MDR (53.5% in 2018–2019, 74.3% in 2019–2020). The mortality rate among MDR and non-MDR isolates were 10.2, and 3.4% (P = 0.28), respectively.

**Table 2:** Prevalence of Gram-negative bacteria isolated from SSIs

Bacteria	Frequency	Percent
*Klebsiella* spp.	23	29.5
*Enterobacter* spp.	22	28.2
*Acinetobacter* spp.	13	16.7
*Escherichia coli*	9	11.5
*Proteus* spp.	5	6.4
*Pseudomonas* spp.	4	5.1
*Citrobacter* spp.	2	2.6
Total	78	100.0
*Enterobacteriaceae*	61	78.2
NFGNB	17	21.8

*NFGNB* non-fermenting Gram-negative bacilli

**Table 3:** Antibiotic resistance pattern of Gram-negative bacteria isolated from SSIs^a^

Class	Antibiotic	*Enterobacteriaceae *(%)	*Pseudomonas* spp. (%)	*Acinetobacter* spp. (%)
Aminoglycosides	Amikacin	25	66.7	100
	Gentamicin	64.5	100	66.7
Carbapenems	Imipenem	46.2	100	100
	Meropenem	65.5	100	85.7
Penicillins	Piperacillin	66.7	33.3	100
Cephems	Ceftazidime	60	50	100
	Cefepime	80	0	100
	Cefixime	80	NT	100
	Ceftriaxone	56.3	NT	100
	Cefoxitin	55.0	NT	100
Fluoroquinolones	Ciprofloxacin	70.6	50	100
Macrolides	Azithromycin	65	NT	NT
Sulfonamides	SXT	25	NT	60
Susceptible strains^b^		3.3	0	0
MDR		67.2	50	46.2

*NT* not tested, *SXT* trimethoprim/sulfamethoxazole, *MDR* multiple drug-resistant

^a^The results presented as percentage of resistant strains for each of the tested antibiotics

^b^The percentage of strains which were susceptible to all of the tested antibiotics

### Discussion

Despite the advances in the management of patients undergoing surgery, SSIs remain a significant risk of surgery [18]. The proper treatment of SSIs relies on early identification of infecting etiology and antibiotic susceptibility patterns. However, because of the potentially life-threatening nature of SSIs, immediate treatment can be precious to save the life of the patients [19, 20]. So that knowing the local epidemiology and antibiotic resistance pattern will help to reduce the risks of treatment failure.

In our study, *Enterobacteriaceae* (78.2%) followed by *Acinetobacter* spp. (16.7%) were the most common isolated GNB from SSIs. Despite the variations based on wound class, surgical procedures studied population, or geographical distribution [21], there are several comparable reports showed that *Enterobacteriaceae* and non-fermenting Gram-negative bacilli (NFGNB) including *Acinetobacter* spp. and *Pseudomonas* spp. are the most prevalent GNB in SSIs [22–27]. However, the hospitalized ward can be a key determining factor for changing the isolation pattern of these bacteria. For instance, in our study, NFGNB mostly isolated from patients in ICUs, while *Enterobacteriaceae* were mostly seen in general wards (Additional file 2). Previously, it has been shown that NFGNB is the main nosocomial pathogen of ICUs [28].

Antibiotic resistance patterns are usually greatly varied, mostly because of differences in the geographical area, type of organisms, and methods used. However, closest to our findings, individual reports from India, Ethiopia, and Rwanda introduced aminoglycosides and carbapenems as the most effective antibiotics against GNB recovered from patients with SSIs [22, 24, 25]. In recent years, the emergence of MDR strains, particularly extended-spectrum beta-lactamases (ESBL) and carbapenemases producing GNB become a growing global problem [29–32]. In our results, the estimated rate of MDR isolates was remarkable (62.8%). Previously, it has been shown that MDR isolates can be associated with different types of SSIs and an increased risk of treatment failure [33, 34].

In summary, a remarkable rate of MDR isolates which showed an increasing trend during recent years is a serious alarm for the management of SSIs caused by GNB. Meanwhile, despite the comparatively stable pattern of causative agents of SSIs at a global scale, the continuous evolution of pathogens in hospital environments necessitates continuous updating on antimicrobial resistance profiles to overcome the risk of treatment failure. Also, more clinical and research attention to patients with diabetes and hypertension who may be at higher risk of infections is recommended.

## Limitations

Our study had several limitations that must be acknowledged. In the present study, we did not explore the microbial spectrum of Gram-positive bacteria as one of the main causes of SSIs. Also, as a limitation of descriptive studies and the unavailability of some information, we were unable to explore the risk of prophylaxis and the emergence of drug-resistant isolates and the risk of postdischarge infections.

## Supplementary information

**Additional file 1.** Distribution of underlying diseases among studied cases.

**Additional file 2.** Distribution of Gram-negative bacteria based on isolation ward.

## Data Availability

The datasets used and/or analyzed during the current study are available from the corresponding author on reasonable request.

## Abbreviations

SSIs: Surgical site infections
MDR: Multiple drug-resistant
GNB: Gram-negative bacteria
CDC: Centers for Disease Control and Prevention
CLSI: Clinical and Laboratory Standards Institute
ICU: Intensive care unit
NFGNB: Non-fermenting Gram-negative bacilli
ESBL: Extended-spectrum beta-lactamases
