# Supplementary material for: Two years study of prevalence and antibiotic resistance pattern of Gram-negative bacteria isolated from surgical site infections in the North of Iran
Source: BMC Res Notes. 2020 Aug 14;13:383. doi: 10.1186/s13104-020-05223-x (PMC7427747; doi:10.1186/s13104-020-05223-x)
Supplement: Supplementary file 1 — Additional file 1. Distribution of underlying diseases among studied cases. [file 13104_2020_5223_MOESM1_ESM.docx]

Additional file 1. Distribution of underlying diseases among studied cases

| **Underlying Diseases** | **Frequency** | **Percent** |
| --- | --- | --- |
| Diabetes | 8 | 10.3 |
| Leg Fracture | 3 | 3.8 |
| Diabetes, CABG | 1 | 1.3 |
| Diabetes, Hypertension | 1 | 1.3 |
| Diabetes, Metastatic Endometrial cancer | 1 | 1.3 |
| Diabetes, CHD, Hyperlipidemia, CVA (old), Hypertension | 1 | 1.3 |
| Diabetes, ESRD , Laminectomy | 1 | 1.3 |
| Hypertension, CHD | 1 | 1.3 |
| Hypertension, CKD | 1 | 1.3 |
| Hypertension, Hyperlipidemia, CABG | 1 | 1.3 |
| Hypertension, Hypothyroidism | 1 | 1.3 |
| Hypertension, Ischemic heart disease, CVA (old) | 1 | 1.3 |
| Craniotomy | 1 | 1.3 |
| Hypothyroidism | 1 | 1.3 |
| Hypothyroidism, HCV | 1 | 1.3 |
| Hysterectomy | 1 | 1.3 |
| Kidney stone | 1 | 1.3 |
| Cervical stenosis | 1 | 1.3 |
| Lumbar disc disease | 1 | 1.3 |
| Neuroblastoma | 1 | 1.3 |
| Psychology disorders | 1 | 1.3 |
| None | 48 | 61.5 |

Abbreviations: Coronary artery bypass grafting (CABG); Coronary heart disease (CHD); Cerebrovascular accident (CVA); Chronic kidney disease (CKD); Hepatitis C virus (HCV)
