# Supplementary material for: Two years study of prevalence and antibiotic resistance pattern of Gram-negative bacteria isolated from surgical site infections in the North of Iran
Source: BMC Res Notes. 2020 Aug 14;13:383. doi: 10.1186/s13104-020-05223-x (PMC7427747; doi:10.1186/s13104-020-05223-x)
Supplement: Supplementary file 2 — Additional file 2. Distribution of Gram-negative bacteria based on isolation ward. [file 13104_2020_5223_MOESM2_ESM.docx]

Additional file 2. Distribution of Gram-negative bacteria based on isolation ward

| **Ward** | ***Acinetobacter* spp.** | ***Citrobacter* spp.** | ***E. coli*** | ***Enterobacter* spp.** | ***Klebsiella* spp.** | ***Proteus* spp.** | ***Pseudomonas* spp.** |
| --- | --- | --- | --- | --- | --- | --- | --- |
| General surgery | 0 | 0 | 1 | 0 | 2 | 4 | 0 |
|  | 0.0% | 0.0% | 11.1% | 0.0% | 8.7% | 80.0% | 0.0% |
| General ICU | 2 | 0 | 1 | 2 | 2 | 0 | 2 |
|  | 15.4% | 0.0% | 11.1% | 9.1% | 8.7% | 0.0% | 50.0% |
| Neurosurgery ICU | 5 | 0 | 4 | 5 | 5 | 0 | 2 |
|  | 38.5% | 0.0% | 44.4% | 22.7% | 21.7% | 0.0% | 50.0% |
| Neurology unit | 1 | 0 | 1 | 1 | 0 | 0 | 0 |
|  | 7.7% | 0.0% | 11.1% | 4.5% | 0.0% | 0.0% | 0.0% |
| NICU | 2 | 0 | 0 | 2 | 2 | 0 | 0 |
|  | 15.4% | 0.0% | 0.0% | 9.1% | 8.7% | 0.0% | 0.0% |
| Orthopaedic | 2 | 2 | 1 | 12 | 11 | 1 | 0 |
|  | 15.4% | 100.0% | 11.1% | 54.5% | 47.8% | 20.0% | 0.0% |
| PICU | 1 | 0 | 1 | 0 | 1 | 0 | 0 |
|  | 7.7% | 0.0% | 11.1% | 0.0% | 4.3% | 0.0% | 0.0% |
